# Upon a Function of the Pancreas but Little Known; the Digestion of Nitrogenous Food

**Published:** 1859-07

**Authors:** 


ART. V.—*Sur une Fonction peu connue du Pancréas; la Digestion des Aliments Azotés.* Par LUCIEN CORVISART, M.D. 8vo., pp. 123. Victor Masson: Paris, 1857–58.

*Upon a Function of the Pancreas but little known; the Digestion of Nitrogenous Food.* By LUCIEN CORVISART.

ACCORDING to the best treatises upon physiology, the functions of the pancreas are limited to the digestion of the non-nitrogenized, or calorifacient food. Upon the starch of vegetable aliments the pancreatic juice acts in a similar manner to the saliva, transforming it first into sugar, and ultimately into lactic acid; upon the fats of animal food it exerts a peculiar emulsyfying power, reducing them to a state of molecular division, which, if it does not put them in a condition to be taken up by the absorbents, is an important step in their digestion. According to the author of the treatise now under notice, the functions of the pancreas are not thus limited to the digestion of non-nitrogenized food; the new function heretofore little known, which he maintains this organ possesses, is the digestion of albuminous food in all its varieties. He does not maintain, however, that the views heretofore held in regard to the action of the gastric juice upon this class of food are incorrect, nor attempt, in showing that these aliments undergo more change in the intestines than has heretofore been believed, to take from the stomach any of the functions now attributed to it. He looks upon the action of the pancreatic juice in the digestion of nitrogenized food rather as supplemental to that of the stomach, it having no effect upon the peptones produced in the stomach, but acting energetically upon that portion of the food which may have escaped gastric digestion, either on account of the excessive quantity taken, or from any defect in the actions of that organ.

The objects of the author have been to ascertain what are the changes induced in plastic, or tissue-making food by the pancreatic juice, and so establish the relations and differences between gastric and intestinal digestion. In pursuing his investigations, he has not been unmindful of the difficulties which attend the subject; these are far greater than those which have been overcome in ascertaining the functions of the stomach; gastric fistulæ afford a ready means for the introduction of any article of food into the stomach in a state of purity; it can remain there unacted upon by anything except the gastric juice, and may be removed at will. But no sooner does the food pass from the stomach, than it meets with the bile and the secretion from the glands of the intestine, as well as the pancreatic juice, the presence of all interfering with our observation of the action of any one of them; moreover, the matters poured into the intestine from the stomach are far from being simple in character. They consist of three different classes, even after the most careful selection of aliment: First, digested food, the product of the action of gastric juice upon the ingesta; second, undigested food, that which has escaped any change in the stomach; third, that portion of the gastric juice which has not acted upon the food and remains pure, or nearly so. The author has also kept in view the fact that *digestion* is something more than mere *solution*, and the product of the experiments made has been carefully tested in every instance and proved to be not only dissolved, but changed. These experiments were of two kinds: upon animals, by ligating the duodenum at its upper and lower extremity, and placing within it various alimentary substances, and by submitting the same articles to artificial digestion with the pancreatic juice, or with a mixture of the pancreatic glands of animals and water. He made use of the principal nitrogenized articles of food in a state of the greatest possible purity; albumen, cooked and raw, fibrin, cellular tissue, muscular tissue, caseine, and gelatine were each made the subject of separate investigations. The greater part of the work is taken up with the relation of these different experiments; we will give here only the most important and most practical of the conclusions at which the author has arrived, referring our readers to the work itself for further information upon details:—

1. The azotized aliments are digested by the stomach; they are also digested by the (secretion from the) pancreas.

2. The pancreas is a supplementary organ to the stomach; its action being added to that of the stomach after copious repasts.

3. The nature of the two digestions is similar in so far as the food acted upon by each of them is transformed into assimilable substances exactly similar, (albuminose or peptone.)

5. When azotized aliment has completely undergone gastric digestion, the pancreatic secretion does not exercise any further influence upon it; does not transfer it into another peptone.

6. It is upon that portion of albuminoid substances which has quitted the stomach without being transformed into albuminose that the pancreatic secretion acts.

7. In certain cases the action of the pancreas can equal that of the stomach.

8. If the quantity of digestive fluids is alone considered, the stomach appears far the more powerful, for the gastric juice is ten times more abundant than the pancreatic; but, on the other hand, the pancreatic juice is ten times richer in ferment, (pancreation.)

9. If the action of the gastric juice is aided by the prolonged stay of the aliment and an intimate mixture with it, the pancréatic secretion enjoys the privilege of acting equally in an alkaline, acid, or neutral state, and three times more rapidly.

10. Everything is arranged in the duodenum so that the pancreatic juice may act as soon as it meets the aliment; in the stomach so that a great portion of the food may be transferred into peptones, and further that the other portion may at least be prepared to undergo pancreatic digestion rapidly.

11. This preparation, which varies with the quality and quantity of the food, and of the gastric juice, etc. etc., consists sometimes in simple imbibition, sometimes in extreme division, sometimes in solution,—the pancreatic digestion, being necessarily rapid, finds in the preparation great assistance. The stomach in this respect playing the same *rôle* which is filled by the teeth in regard to gastric digestion.

12. However, the pancreatic juice is capable of digesting aliments which have not undergone this preparation. Thus, albuminous matters, in a crude state, placed directly in the duodenum, are there completely and perfectly digested; the process is only somewhat slower. The pancreatic juice does not need the addition of the bile or the intestinal secretion to give it digestive powers. Practiced artificially in the laboratory with the juice, or with pancreatine, the digestion of azotized aliments takes place the same as in the duodenum itself.

13. When the gastric or pancreatic juices are separated and act successively, each exerts its power fully, and the quantity of albuminose produced can thus be doubled.

14. But it is remarkable that if these two digestive ferments meet in a state of purity, the two digestions do not proceed so freely; instead of the digested product being doubled by this union, it may be reduced to nothing, for in these unnatural circumstances the pepsine and the pancreatine destroy each other's power.

15. In the normal state, nature prevents this conflict by three means: 1. The pylorus, which separates the two ferments. 2. In gastric digestion the pepsine is exhausted and neutralized in forming the pepsine. 3. The bile, as shown by Pappenheim, neutralizes the activity of (that portion of) the gastric ferment, (which has not acted upon the aliment.)

16. The bile does not precipitate the peptone produced by the stomach so that digestion is destroyed and must recommence; it is the bile itself which is precipitated by the acid of the gastric secretion or of the chyme.

17. The kind of azotized aliment used has much influence upon the quantity of peptone which the two digestions can successively produce. Thus, while musculine and caseine furnish nearly thirty grammes of perfect peptone, an equal quantity of albumen or of gelatinous tissues furnish scarcely fifteen grammes.

18. Both digestions, pancreatic as well as gastric, destroy the characteristic properties of the diverse albuminoid substances; they liquefy the insoluble, take from albumen its coagulability, from caseine the property of forming curds, from gelatine that of forming jelly, and from musculine that of giving a precipitate with chloride of sodium; finally, they transform them all into albuminose or peptones.

21. The peptones have for characteristics—solubility in water, acid, neuter or alkaline, which assures an easy circulation through the system; heat does not coagulate them; the acetate of lead does not precipitate the greater part of them; they are, in general, less liable than the azotized alimentary matters to form insoluble metallic combinations.

22. The peptones form a class as well characterized as the albuminous substances; it is evident, however, that science will hereafter determine their natures far more accurately than has yet been done.

COROLLARIES, OR PATHOLOGICAL INDUCTIONS.

(*a*) It is almost certain that there exists, as regards albuminous aliment, a duodenal dyspepsia, caused by the vitiation, the insufficiency, or the absence of the pancreatic secretion, the symptoms of which only appear after the third or fourth hour of digestion, and is accompanied with more sensation than gastric dyspepsia. In these cases of duodenal indigestion the administration of pancreatine is indicated.

(*b*) A secondary duodenal dyspepsia may arise from want of division, in the stomach, of those articles of food not transformed into peptones. Pancreatic digestion then proceeds more slowly, as does the gastric when the teeth do not properly perform their duty. This pancreatic indigestion is to be cured by appropriate treatment of the primitive gastric dyspepsia.

(*c*) Another form of secondary duodenal dyspepsia may arise from a superabundance of gastric juice, or from insufficiency of the pyloric orifice; in both these cases the gastric juice reaches the duodenum in a state of purity, and destroys the power of the pancreatic secretion.

(*d*) A third form of secondary duodenal indigestion may be caused by an insufficient quantity of bile, whereby the gastric juice which passes into the intestine is not neutralized, and the same evil effects are produced as in the two preceding cases.

(*g*) Bile, when it reaches the stomach by the pylorus, or is administered by the mouth, destroys the activity of the gastric juice in that organ. Hence bile may be employed to obviate the evils arising from a superabundance of gastric juice.

(*h*) From equal weights of azotized aliments, and with equal digestive powers, unequal quantities of peptones are produced, according to the nature of the aliment ingested. It is clearly an error, therefore, to estimate the nutritious quality of food merely according to the amount of nitrogen it contains.

(*i*) When it is more urgent to calm the pain and avoid the revolt of the digestive organs than to sustain the muscular force, such food should be given as will dissolve most rapidly and most completely, whatever may be the quantity of peptone it will furnish.

(*j*) But when the most urgent necessity is to support the muscular strength, we should make choice of those aliments which, with equal digestive force, will furnish the greatest quantity of peptone, although they may be more slowly dissolved and digested.

(*k*) He who digests only with one organ (pancreas or stomach) is placed upon half-rations of peptone, the same as one who eats only albumen or gelatinous tissues, which furnish but half as much peptone as caseine or muscular tissue, is upon half rations, and is thus but half nourished. * * * * *

(*l*) We should avoid giving only one kind of azotized food continuously, not only because one kind alone is insufficient to repair the variety of organs in the body, but because a single kind of food, when given exclusively and continuously, after about eight days ceases to excite the secretion of gastric juice, and no longer undergoes gastric digestion.

However carefully the author may have performed his experiments, and however well convinced he may be of the truth of the propositions he has laid down, the whole ground must be gone over again by other and competent investigators, before the profession will look upon his propositions as established. We willingly bear testimony to the careful manner in which he has pursued his investigations, and nothing is apparent in his work but a sincere desire to establish the truth, although some of his views clash with those of Bernard and other eminent physiologists; at the same time, we think there is one important point omitted in his experiments, which is the exclusion of all aliment except nitrogenized. Granting that the pancreatic secretion acts as he shows it to do upon albuminous food, will it continue thus to act when non-nitrogenous aliment is also present? Does it assist in the digestion of albuminous matters at the same time it is forming an emulsion with fats, and acting upon starch? We should have preferred the work had it contained a series of experiments designed to elucidate these questions.

We have called attention to the subject of intestinal digestion as one of great interest to physiologists, and of great importance to practical physicians; it is yet far from being fully understood, and offers a fair field of labor for patient experimenters and investigators. Those who would read some new views upon the whole subject of "Food and Drink," will find a series of articles, evidently written by a master-hand, in *Littel's Living Age* of last year, republished from *Blackwood's Magazine*.

